# Early diagnosis for the onset of peri-implantitis based on artificial neural network

**DOI:** 10.1515/biol-2022-0691

**Published:** 2023-08-31

**Authors:** Wanting Fan, Jianming Tang, Huixia Xu, Xilin Huang, Donglei Wu, Zheng Zhang

**Affiliations:** Department of Stomatology, Shenzhen People’s Hospital, Shenzhen, Guangdong, China; Department of Obstetrics, Shenzhen People’s Hospital, Shenzhen, Guangdong, China

**Keywords:** peri-implantitis, early diagnosis, machine learning, artificial neural network

## Abstract

The aim of this study is to construct an artificial neural network (ANN) based on bioinformatic analysis to enable early diagnosis of peri-implantitis (PI). PI-related datasets were retrieved from the Gene Expression Omnibus database. Differentially expressed genes (DEGs) and functional enrichment analyses were performed between PI and the control group. Furthermore, the infiltration of 22 immune cells in PI was analyzed using CIBERSORT. Hub genes were identified with random forest (RF) classification. The ANN model was then constructed for early diagnosis of PI. A total of 1,380 DEGs were identified. Enrichment analysis revealed the involvement of neutrophil-mediated immunity and the NF-kappa B signaling pathway in PI. Additionally, higher proportion of naive B cells, activated memory CD4 T cells, activated NK cells, M0 macrophages, M1 macrophages, and neutrophils were observed in the soft tissues surrounding PI. From the RF analysis, 13 hub genes (ST6GALNAC4, MTMR11, SKAP2, AKR1B1, PTGS2, CHP2, CPEB2, SYT17, GRIP1, IL10, RAB8B, ABHD5, and IGSF6) were selected. Subsequently, the ANN model for early diagnosis of PI was constructed with high performance. We identified 13 hub genes and developed an ANN model that accurately enables early diagnosis of PI.

## Graphical abstract

**Figure:**
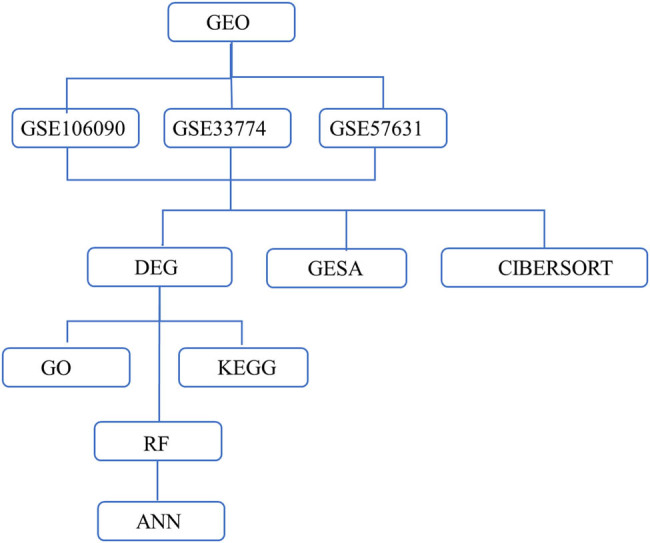

## 1 Introduction

Dental implants afford great convenience to the treatment of missing teeth [1], which show great advantage in restoring functions and esthetics. However, the osseointegration of dental implants can be compromised by biological complications. One such complication is peri-implantitis (PI), which refers to the inflammation and tissue damage surrounding the implants. It is characterized by mucosal inflammation and loss of supporting bone around the implant [2]. Global implant cases have shown a high prevalence of PI, accounting for approximately 22% [3]. Currently, clinicians rely on probing and radiological examinations to diagnose PI in a clinical setting [4]. However, these methods can only detect and diagnose PI after clinical manifestations and signs have already occurred. Therefore, there is a need to identify molecular markers for PI using non-invasive methods. A recent report discovered that cyclophilins, which are increased in the peri-implant crest fluid of PI at week 2, can serve as early biomarkers for the diagnosis of PI, while the loss of supporting bone can be identified through radiological images at week 4 [5]. Hence, investigating and screening early biomarker can support the early intervention or prevention of PI. In addition, the treatment for PI is challenging. In addition, surgical treatment for PI can contribute to bone loss and post-surgical mucosal recession, which compromises the esthetic appearance [4,6]. Therefore, early diagnosis of PI is imperative. With the development of regenerative medicine, identification of hub genes related to PI may provide new information for gene-based therapies in the future [7]. In this study, we aimed to identify the hub genes associated with PI and construct an artificial neural network (ANN) model for the diagnosis of PI based on bioinformatic analysis and machine learning techniques.

## 2 Methods and materials

### 2.1 Data collection and cleaning

The gene expression matrix of PI datasets was obtained from Gene expression omnibus database (https://www.ncbi.nlm.nih.gov/geo/). A total of three datasets were enrolled in this study, including GSE106090, GSE33774, and GSE57631. Nineteen gingival tissues from patients with PI and 16 gingival tissues from healthy control were collected. After standardization and normalization, the datasets were merged and batch effects were removed using the R SVA package.


**Informed consent:** Informed consent has been obtained from all individuals included in this study.
**Ethical approval:** The research related to human use has been complied with all the relevant national regulations, institutional policies and in accordance with the tenets of the Helsinki Declaration, and has been approved by the authors’ institutional review board or equivalent committee.

### 2.2 Identification of differentially expressed genes (DEGs) in PI

Differential expression analysis of gingival tissues between PI and control were performed with R limma package. Genes were considered differentially expressed if they had a false discovery rate (FDR) <0.05 and a |fold change| >1.5. The DEGs expression between the PI and control groups were visualized using a heat map and a volcano plot.

### 2.3 Functional enrichment of DEGs between PI and control group

To further estimate the functional enrichment of DEGs between PI and control group, gene ontology (GO) and Kyoto Encyclopedia of Genes and Genomes (KEGG) analysis were conducted with the R package (clusterProfiler) [8]. The GO (https://geneontology.org/) enrichment analysis comprises three components, including biological process (BP), molecular function (MF), and cellular component (CC). The KEGG (https://www.genome.jp/kegg/) is available for predicting the functions and utilities of the biological system based on the DEGs. The *P* value less than 0.05 was considered significantly difference. In addition, gene set enrichment analysis (GSEA) [9] was performed to annotate the enriched signaling pathway with GSEA software between the PI and control group. The hallmark gene sets database was downloaded from Molecular Signatures Database (h.all.v7.4.symbols.gmt) [10]. The maximum gene set size in GSEA analysis was set to 5,000 and minimum gene set size was set to 5. A *P-*value less than 0.05 and FDR less than 0.25 were considered statistically significant.

### 2.4 Immune cells infiltration between PI and control tissues based on CIBERSORT

To further evaluate the difference of immune microenvironment in peri-implant tissues between the PI and the control group, the CIBERSORT (http://cibersort.stanford.edu/) was applied to calculate 22 leukocytes infiltration in peri-implant tissues with deconvolution method [11]. The 22 leukocyte subsets include plasma cells, naïve and memory B cells, NK cells, myeloid subsets, and seven T cell types.

### 2.5 Identification of hub genes with random forest (RF) classification

RF classification was applied to select the hub genes from the 1,380 DEGs using the randomForest package [12]. The number of decision trees was set to 500 and the error rate was calculated for each forest. The number of optimal trees was determined based on the lowest error rate. Hub genes were evaluated by mean decreased Gini coefficient. Genes with mean deceased Gini coefficients greater than 0 were selected as hub genes. A heatmap was generated to visualize the expression of the hub genes.

### 2.6 Construction of diagnostic model for PI based on ANN

After normalization of the expression profiles, the selected hub genes were applied to construct the ANN based on R package (neuralnet). The merged dataset was randomly divided into a training set and a test set using the Caret package. The training set was used to calculate the weights for the hub genes in the ANN model, while the test set was used to evaluate the generalization ability of the model. The ANN model consisted of five hidden layers. The receiver operating characteristic (ROC) curve was employed to assess the sensitivity and specificity of the diagnostic ANN model for PI.

## 3 Results

### 3.1 Identification of DEGs between PI and control

**Figure 1:**
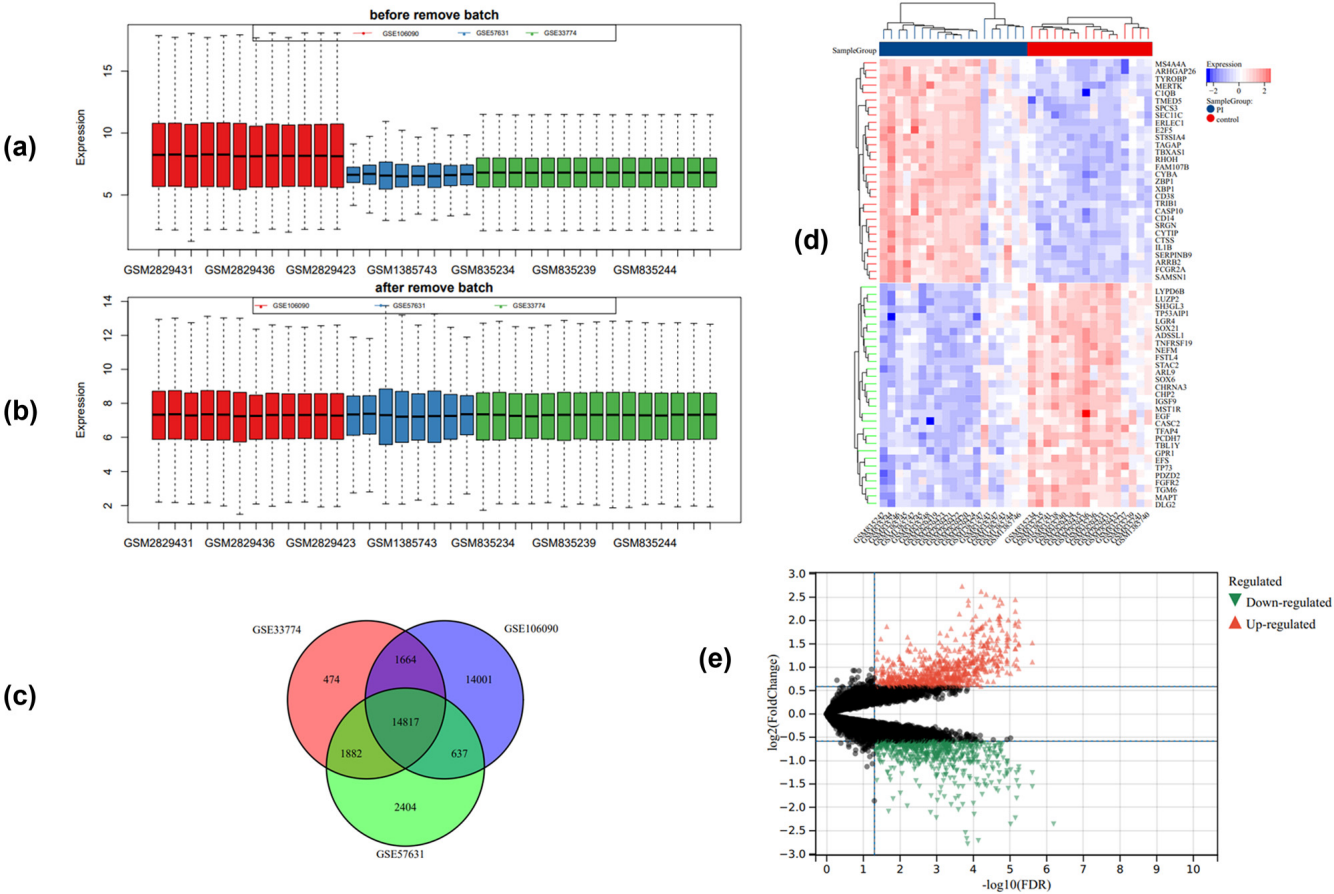
Data cleaning, merging, and identification of DEGs. (a) Bar plot of gene expressions in three datasets before batch effect removal. (b) Bar plot of gene expression in three datasets after batch effect removal. (c) Venn diagram of the intersection of genes from three datasets. (d) Heatmap of top 30 DEG expressions between PI and control groups. (e) Volcano plots of downregulated and upregulated genes between PI and control groups.

After removing the batch effect, the expression of genes in the merged datasets was normalized (Figure 1a and b). A total of 14,817 genes were co-expressed in the 3 datasets (Figure 1c). Among them, 756 upregulated DEGs and 624 downregulated DEGs were identified between the PI and control groups (Figure 1d and e).

### 3.2 Identified functional enrichment of DEGs between PI and control group

**Figure 2:**
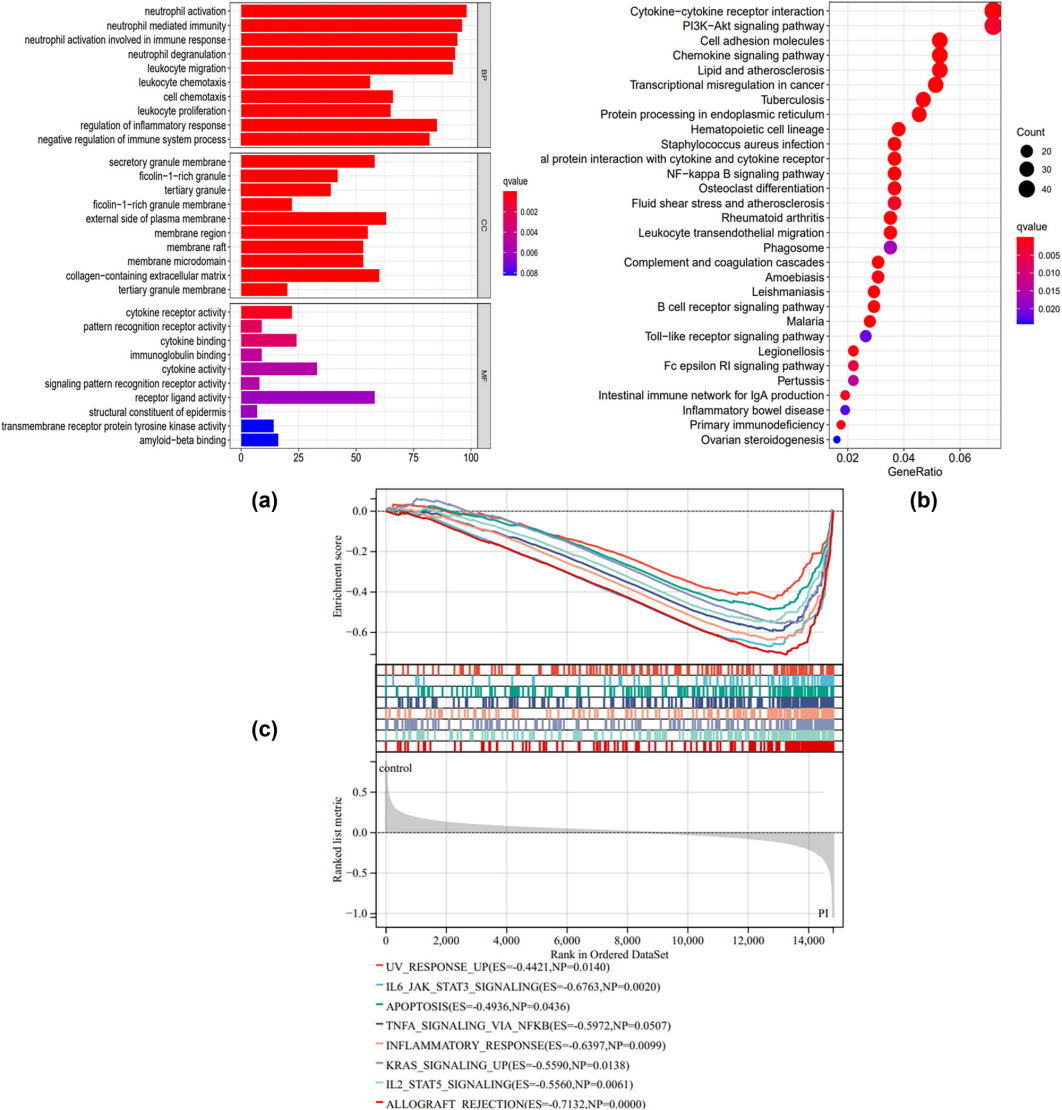
Functional enrichment of DEGs between PI and control group. (a) Bar plot of GO enrichment analysis. The abscissa axis indicates gene number. The ordinate indicates enriched biological structure and function. BP, biological process; MF, molecular function; CC, cellular component. (b) Bubble chart of the KEGG enrichment analysis. The abscissa axis indicates the gene ratio. The ordinate indicates an enriched signaling pathway. (c) GSEA analysis of DEGs between the PI and control group.

The BP component in GO analysis indicated that neutrophil activation, neutrophil mediated immunity, leukocyte migration, and negative regulation of immune system process were the main BPs in the advance of PI. In addition, external side of plasma membrane, collagen-containing extracellular matrix, and secretory granule membrane were enriched in the CC component. Moreover, the MF component was concentrated on receptor ligand activity, cytokine activity, and cytokine receptor activity (Figure 2a). The result of KEGG analysis indicated that the cytokine−cytokine receptor interaction, PI3K−Akt signaling pathway, nuclear factor (NF)-kappa B signaling pathway, fluid shear stress, and atherosclerosis related signaling pathway, osteoclast differentiation, and leukocyte transendothelial migration signaling pathway were enriched based on the DEGs between the PI and control groups (Figure 2b). Moreover, the GSEA results demonstrated that various signaling pathways were positively correlated with the occurrence of PI. These pathways included the UV response signaling pathway, IL6/JAK/STAT3 signaling pathway, apoptosis signaling pathway, TNF-α signaling pathway via NF-Kappa B, inflammatory response signaling pathway, KRAS signaling pathway, IL2/STAT5 signaling pathway, and allograft rejection signaling pathway (Figure 2c).

### 3.3 Identified 22 infiltrated immune cells between PI and control tissues

**Figure 3:**
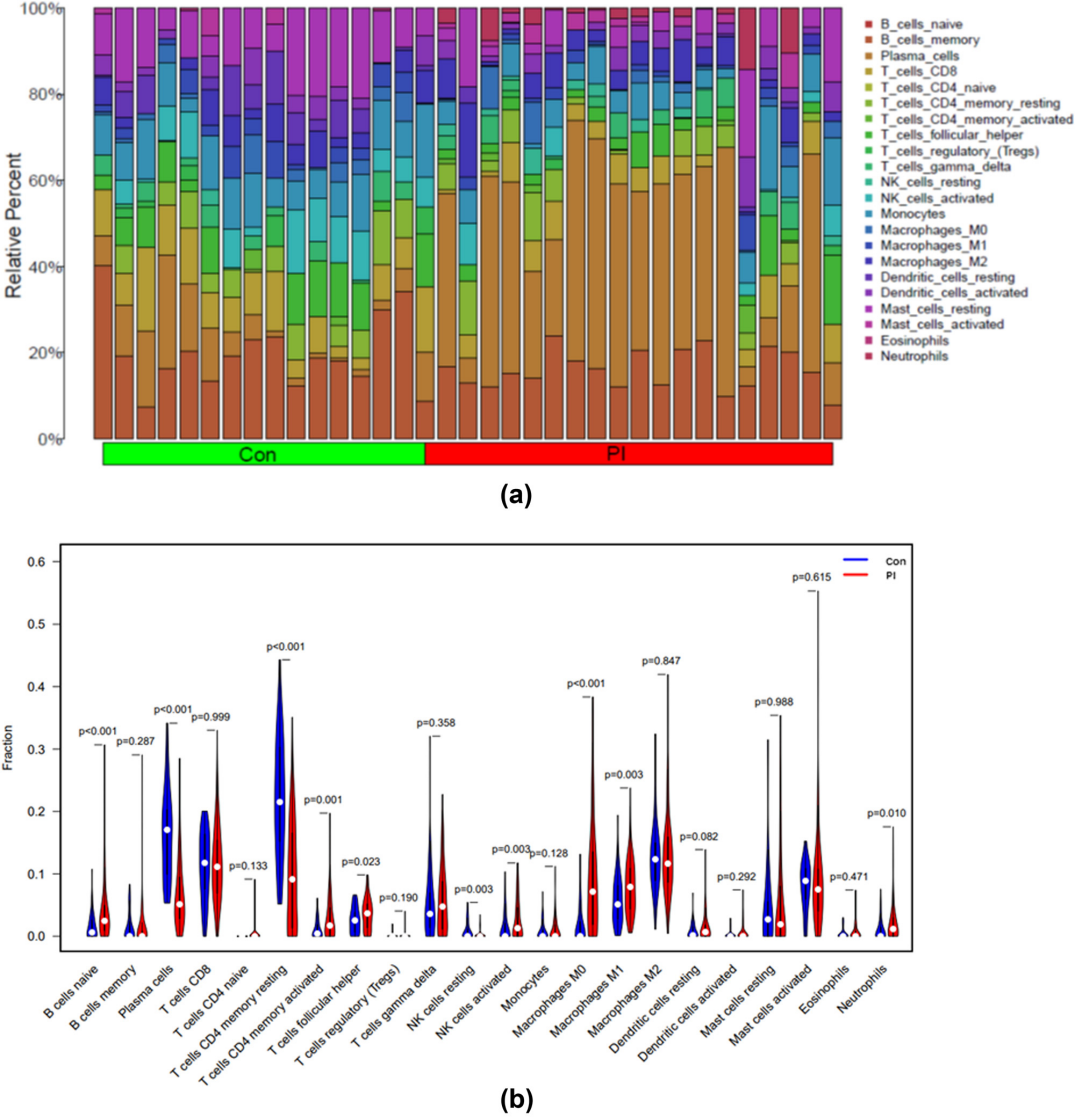
Identified 22 immune cell infiltrations between PI and control tissues. (a) Bar plot of proportion of 22 leukocytes in gingival tissues. (b) Violin plot of proportion of 22 leukocytes in gingival tissues between PI and control group.

The CIBERSORT results indicated a predominant infiltration of CD8 T cells, resting memory CD4 T cells, M0 macrophages, M1 macrophages, M2 macrophages, and activated mast cells in PI tissues (Figure 3a). In contrast, gingival tissues in the healthy control group were predominantly infiltrated by plasma cells, CD8 T cells, and resting memory CD4 T cells infiltration. Compared with the control group, soft tissues surrounding the PI exhibited a higher proportion of naive B cells, activated memory CD4 T cells, activated NK cells, M0 macrophages, M1 macrophages, and neutrophils, while lower level of plasma cells, resting memory CD4 T cells, and resting NK cells were identified (*P* < 0.05) (Figure 3b).

### 3.4 Identification of hub genes for PI based on RF classification

**Figure 4:**
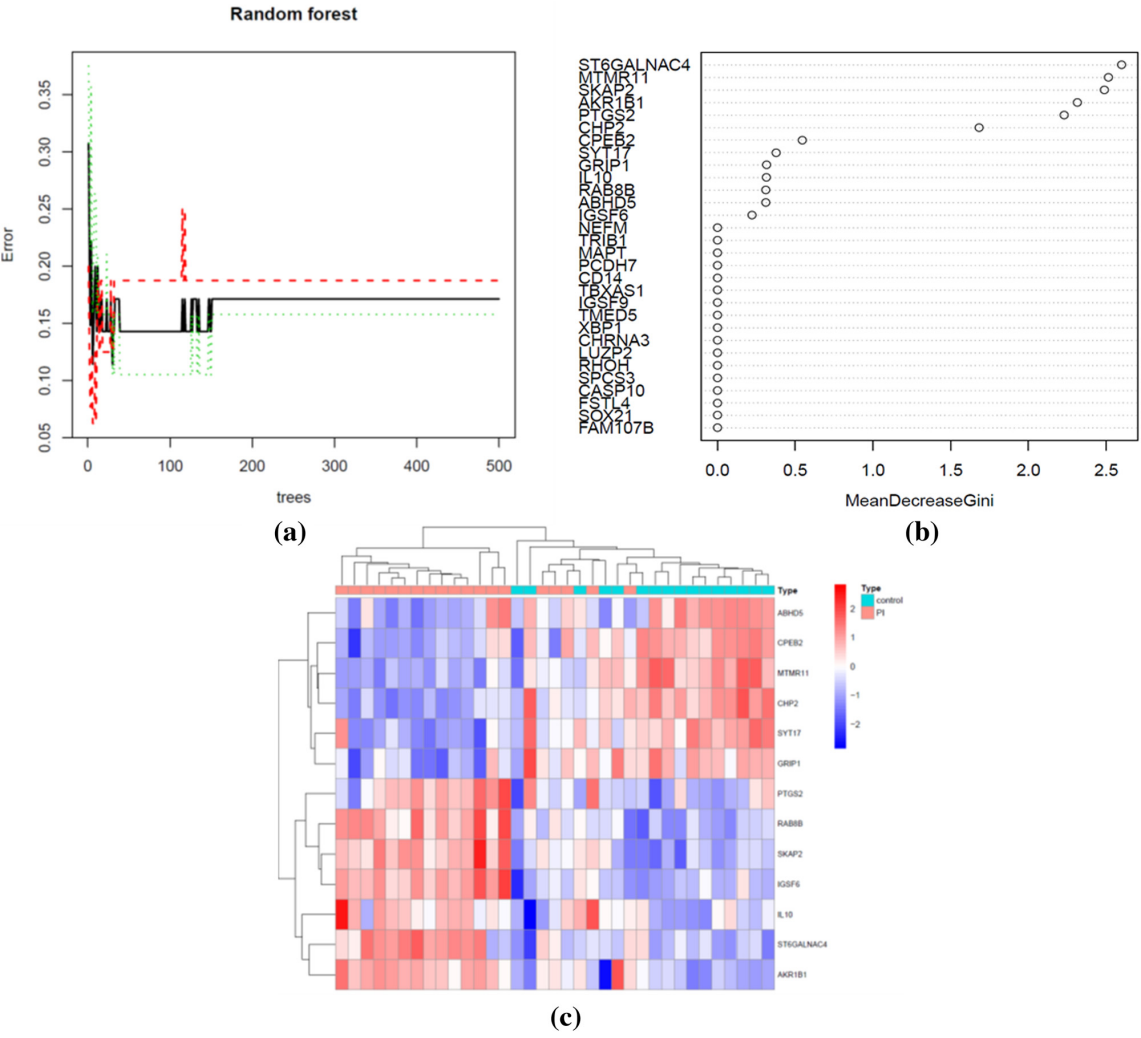
Construction of RF classification. (a) The influence of the number of decision trees on the error rate. The *x*-axis indicates the number of decision trees, and the *y*-axis indicates the error rate. (b) The importance of DEGs based on mean decreased Gini coefficient in the RF model. (c) Heatmap of 13 hub genes expressed in PI and control tissues.

The RF classification was constructed with an optimal number of 12 decision trees (Figure 4a). A total of 13 hub genes were selected from the list of DEGs based on the mean decreased Gini coefficient. These hub genes included ST6GALNAC4, MTMR11, SKAP2, AKR1B1, PTGS2, CHP2, CPEB2, SYT17, GRIP1, IL10, RAB8B, ABHD5, and IGSF6 (Figure 4b). The heatmap revealed upregulation of ST6GALNAC4, AKR1B1, PTGS2, RAB8B, SKAP2, IGSF6, and IL-10 and downregulation of ABHD5, CPEB2, MTMR11, CHP2, SYT17, and GRIP1 in the PI group (Figure 4c).

### 3.5 Construction of PI diagnostic model based on ANN

**Figure 5:**
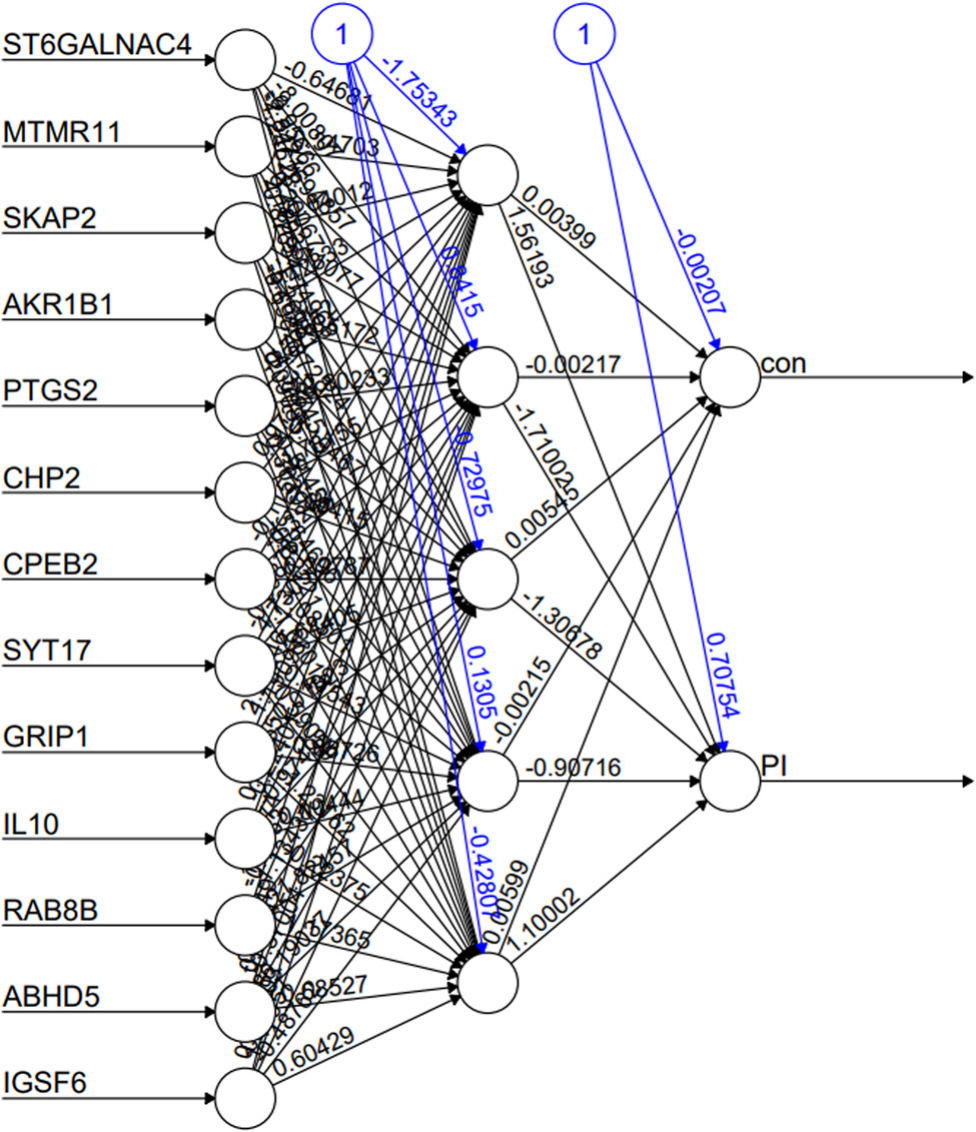
Early diagnosis of PI based on ANN.

**Figure 6:**
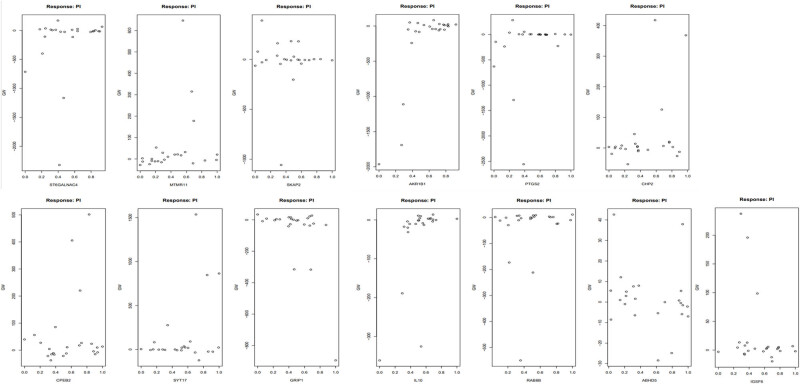
Scatter plots of generalized weights in 13 genes.

The ANN employed for the diagnosis of PI consisted of 13 input layers, 5 hidden layers, and 2 output layers (Figure 5) The ANN underwent 423 iterations, with a loss function value of 0.008 and a maximum weight adjustment of 0.009. The intercept terms for the first hidden layers were sequentially −1.75, 0.84, −0.73, 0.13, and −0.43. The connecting weighting coefficients between 13 hub genes and the first hidden layers were as follows: −0.65 (ST6GALNAC4), −0.35 (MTMR11), −0.64 (SKAP2), 1.43 (AKR1B1), −0.78 (PTGS2), −0.97 (CHP2), −0.12 (CPEB2), −0.31 (SYT17), 2.24 (GRIP1), 0.08 (IL10), −1.67 (RAB8B), −0.32 (ABHD5), and 0.37 (IGSF6). In addition, a nonlinear correlation was observed between the generalized weight and impotence of 13 hub genes (Figure 6). The areas under the ROC curves (AUC) for the test set was 1, indicating a high robustness of the ANN for PI diagnosis (Figure S1).

## 4 Discussion

The fast-developing machine learning in high-throughput omics data facilitated the disease of early detection and diagnosis based on molecular profile matrices [13]. In recent years, artificial intelligence (AI) technologies have made great advances in various dental specialties. ANN, as one of the AI models, are extensively adopted in the detection and diagnosis of dental caries, salivary gland diseases, and maxillofacial cysts [14]. ANN, a type of data structure inspired by networks of biological neurons organized in layers, is widely regarded as the most efficient model for pattern recognition through automated processes [15]. The diagnostic performance of ANN has been proven to be superior to clinicians or at least comparable to that of clinicians [16]. A recent study has constructed a diagnostic ANN model with high accuracy for periodontitis [17]. Utilizing ANN applications can assist in personalized clinical decision-making, including early diagnosis, therapeutic strategy development, and prognostic assessment.

Given the increasing demand for dental implants, it is imperative to develop techniques for early diagnosis in PI. Recent researchers have constructed a risk-grading model for PI using machine learning algorithm. These models classify PI into high-risk and low-risk groups based on immune profiling, which is relevant to clinical outcomes and microbial composition [4]. In addition, risk assessment for PI using demographic data based on RF model show the best predictive performance than logistic regression and support vector machines [18]. RF predicted nearly 70% of PI cases [18]. In this study, we first constructed an ANN model with high performance for PI diagnosis based on transcriptome data, which can facilitate the clinical decision making.

Not only inflammation but also the implant itself was responsible for the alteration of immune microenvironment in gingival tissues surrounding the implant [19]. PI tends to provoke more excessive host immune responses. A recent study has identified higher macrophages infiltration in PI tissues compared to periodontitis and health control in the animal model, which was consistent with our results [19]. Macrophage polarization was involved in the development of PI lesions [20,21]. A higher level of M1 inflammatory phenotype and an increased M1/M2 ratio were observed in advanced PI tissues, which revealed an inflammation around dental implants [20]. Moreover, macrophage polarization may delay the peri-implant tissues healing after surgical therapy of PI [22]. Gingival tissues surrounding PI were characterized by an increase in the number of CD4+ T lymphocytes and B lymphocytes [23,24]. We also confirmed an increase in activated NK cells and neutrophils in tissues surrounding PI, which might regulate the hypoxia microenvironment via HIF-1α [25]. However, another research found that the immune profile with elevated M1/M2-like macrophage ratios and lower B-cell infiltration predict a lower risk for the occurrence of PI through machine learning [4].

Routine monitoring of dental implant plays an important role in preventing the occurrence of PI. However, currently there are still no standard diagnostic test for PI. Our study identified 13 hub genes of PI, which could potentially serve as key biomarkers for point-of-care testing for this condition [26]. Among the hub genes, ST6GALNAC4, a glycosyltransferase gene, plays an important role in catalyzing the transfer of sialic acid residues to the terminal positions of the glycoprotein and glycolipid carbohydrate groups. This is the first study that associates ST6GALNAC4 with PI. However, research on ST6GALNAC4 (gene or enzyme) is limited at present. A recent study found that patients with acute coronary syndrome had a lower expression of ST6GALNAC4 [27]. In addition, ST6GALNAC4 was found to be responsible for the invasion and metastasis in follicular thyroid cancer [28]. Interleukin (IL)-10 was closely related to the development of PI [29,30]. Several published studies have reported an increase in IL-10 expression [31,32,33]. However, another study demonstrated a negative correlation between IL-10 levels and the peri-implant probing depth in PI [30]. Consistent with the findings of Bressan et al. [34], increased expression of prostaglandin-endoperoxide synthase 2 (PTGS2) was observed in PI tissues. The protein-coding gene IGSF6, belonging to the immunoglobulin superfamily member 6, is involved in the regulation of inflammatory bowel disease [35]. Multiple hub genes were associated with the regulation of inflammation and immune metabolism, suggesting a significant role in the onset of PI. SKAP2, a gene encoding Src kinase-associated phosphoprotein 2, is responsible for B-cell and macrophage adhesion processes. Aldose reductase (AKR1B1) is a NADPH-dependent aldo-keto reductase involved in polyol biosynthesis [36]. However, the reduction of aldehydes which was catalyzed by AKR1B1 enabled the activation of intracellular inflammatory via the NF-κB signaling pathway [37]. The NF-κB signaling pathway in macrophages is considered critical for the pathological mechanisms of PI. The abhydrolase domain containing 5 (ABHD5), an activator of triglyceride hydrolysis in macrophages, plays a crucial role in inflammation resolution and metabolic reprogramming in cancer. ABHD5 suppressed the excessive production of reactive oxygen species and pro-inflammatory cytokines by inhibiting the activation of NLRP3 inflammasome in macrophages [38]. In addition, ABHD5 exhibits low expression in migratory tumor-associated macrophages. Recent research has indicated that ABHD5 in macrophages inhibited the production of matrix metalloproteinases (MMP) in an NF-κB-dependent manner, effectively reducing colorectal cancer metastasis [39]. Similarly, CHP2 was identified to reduce oxidative damage and restrain the activation of NF-κB-p65 [40]. GRIP1, an essential regulator of immunometabolism, engaged in modulating the macrophage polarization and maintaining the metabolic homeostasis [36].

The primary limitation of this study is the small sample size. Machine learning algorithms heavily rely on large datasets for learning and training. Limited data in training set and test set could increase the risk of overfitting in the ANN model [41]. Furthermore, the study did not have access to clinical and imaging characteristics from public databases. Only the PI transcriptome was included in construction of diagnostic model, neglecting the involvement of genetic, epigenetic, and environmental factors in the onset of PI [42]. Notably, hypermethylation and titanium dissolution particles have been linked to the development of PI [42]. Therefore, the value of ANN diagnostic model for PI requires further validation using larger sample sizes and multiple omics data.

## 5 Conclusion

In summary, we identified 13 hub genes and constructed the ANN model for the early diagnosis of PI, which is imperative for personalized clinical decision making. Further works are needed to verify those genes’ functions for PI.

## Supplementary Material

Supplementary Figure
