# Supplementary figures and images for: Early diagnosis for the onset of peri-implantitis based on artificial neural network
Source: Open Life Sci. 2023 Aug 31;18(1):20220691. doi: 10.1515/biol-2022-0691 (PMC10476483; doi:10.1515/biol-2022-0691)

# Supplementary material

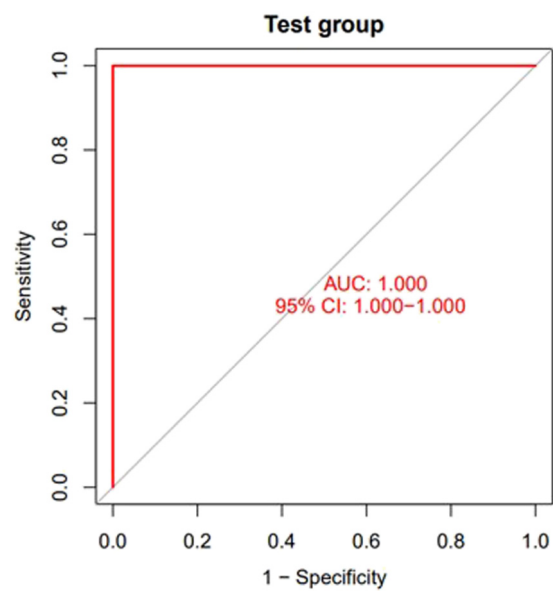

**Figure S1:** The ROC curves for the ANN for PI diagnosis.

Supplement: Supplementary Figure [file biol-2022-0691-sm.pdf]
